# Positional dependence of activity determination in single photon emission computed tomography

**DOI:** 10.1097/MNM.0000000000001034

**Published:** 2019-05-24

**Authors:** Emlyn Price, Jill Tipping, David M. Cullen, Nick Calvert, David Hamilton, Emma Page, Sophia Pells, Ben Pietras, Andrew P. Robinson

**Affiliations:** a Schuster Laboratory, School of Physics and Astronomy, The University of Manchester; b Christie Medical Physics and Engineering (CMPE), The Christie NHS Foundation Trust, Manchester; c National Physical Laboratory, Teddington, UK

**Keywords:** molecular radiotherapy, quantitative imaging, radionuclide imaging, single photon emission computed tomography computed tomography

## Abstract

Accurate image quantification requires accurate calibration of the detector and is vital if dosimetry is to be performed in molecular radiotherapy. A dependence on the position of calibration has been observed in single photon emission computed tomography images when attenuation correction (AC) and scatter correction are applied. This work investigates the origin of this dependence in single photon emission computed tomography scans of phantom inserts filled with ^177^Lu solution. A 113 ml sphere and inserts representing a mathematical model of a spleen and an anatomical model of a patient spleen were imaged at the centre and edge of elliptical phantoms. For these inserts, the difference in calibration factor between the positions was around 10% for images reconstructed with AC and triple energy window scatter correction. A combination of experimental imaging and Monte Carlo simulation was used to isolate possible causes due to imaging or reconstruction in turn. Inconsistent application of AC between different reconstruction systems was identified as the origin of the positional dependence.

## Introduction

The 3D quantification of the activity distribution in a patient is essential if meaningful dosimetry is to be carried out in nuclear medicine therapies. The counts extracted from reconstructed single photon emission computed tomography (SPECT) images must be related to this activity distribution. The relationship between counts and activity depends on the scanner used, the corrections applied during image reconstruction and the volume of interest (VOI) [1]. The effect of position in the scanner field of view on the calibration factor has been previously observed for ^131^I and ^177^Lu [2]. In a study by Wevrett *et al.* [2], spheres were displaced by up to 12.8 cm from the centre of an elliptical Jaszczak phantom. The spheres were imaged on GE Infinia Hawkeye, GE Discovery 670 and Siemens Symbia T cameras. The images were then reconstructed with native and vendor-neutral software. The cause of the positional dependence of the calibration factor was suggested to be the depth-dependent spatial resolution (DDSR) of SPECT scanners [2]. However, data were presented in Table 1 of Wevrett *et al.* [2] for one scanner and reconstruction platform combination, a Siemens Symbia with a vendor neutral reconstruction, in which positional dependence was not apparent. No resolution recovery was used for the data in Table 1 of Wevrett *et al.* [2]. The lack of positional dependence for one system when resolution recovery is not used suggests that the cause may be related to the choice of reconstruction parameters rather than the DDSR.

**Table 1:**
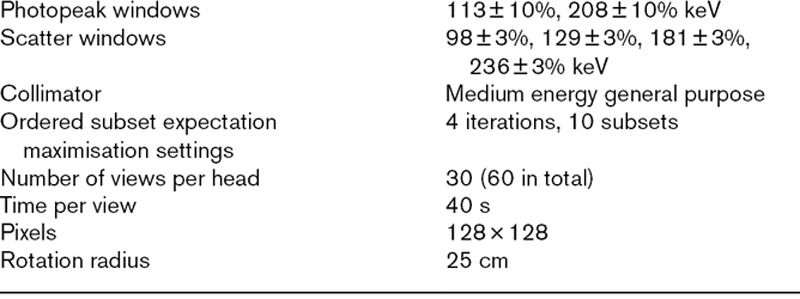
Acquisition settings for the single photon emission computed tomography scans of inserts filled with ^177^Lu

The data presented in this work reveal a positional dependence when inserts within a phantom are filled with ^177^Lu-Dotatate solution and displaced similarly to those in Wevrett *et al.* [2]. It is important to note that if a phantom is positioned centrally then a radial displacement of an insert has two effects: the distance from the insert to the detector changes and the insert is closer to the edge of any attenuating material in the phantom. A combination of experimental imaging and Monte Carlo simulation was used to isolate possible causes of the positional dependence. In this work, the potential causes considered were:

(1) DDSR of the collimator.
(2) Signal processing of the PMT data.
(3) The choice of reconstruction algorithm implementation and attenuation correction (AC) method.
(4) The method of scatter correction used.

Triple energy window (TEW) scatter correction was compared to Monte Carlo scatter correction for point sources, spheres and cylinders in [3] for ^131^I, ^177^Lu and ^188^Re using a Siemens Symbia scanner. Zhao *et al.* [3] found that a planar scan of a point source can be used to calculate the calibration factor for TEW corrected SPECT scans and that TEW scatter correction overestimates scatter for inserts surrounded by a cold background. This work additionally examines the effect of position on calibration factor.

## Methods

Experimental scans of the following inserts were performed:

(1) A commercially available 113 ml sphere.
(2) A commercially available 16 ml sphere.
(3) A 3D printed model of the Cristy and Eckerman (C&E) spleen, described by Robinson *et al.* [4].
(4) A 3D printed patient spleen, described by Price *et al.* [5].

All the scans were performed on a GE Infinia Hawkeye 4 (GE Healthcare, Chicago, Illinois, USA) SPECT/computed tomography (CT) scanner. Images were acquired for the 113 keV (EM1) and 208 keV (EM2) ^177^Lu photopeaks, with adjacent scatter windows [6]. The acquisition settings are shown in Table 1. Low dose hybrid CT scans were acquired to allow CT-based AC and delineation of the insert boundaries. All the images were reconstructed using ordered subset expectation maximisation algorithms. No post filter or resolution recovery process was applied. Two reconstruction systems were used, GE Xeleris (version 3; GE Healthcare, Chicago, Illinois, USA), referred to as ‘vendor specific’ and Hermes HybridRecon (version 2; Hermes Medical Solutions AB, Stockholm, Sweden), referred to as ‘vendor neutral’. The standard technique used for calibration at the Christie is to image a large uniform cylinder, as suggested by Dewaraja *et al.* [1].

**Fig. 1:**
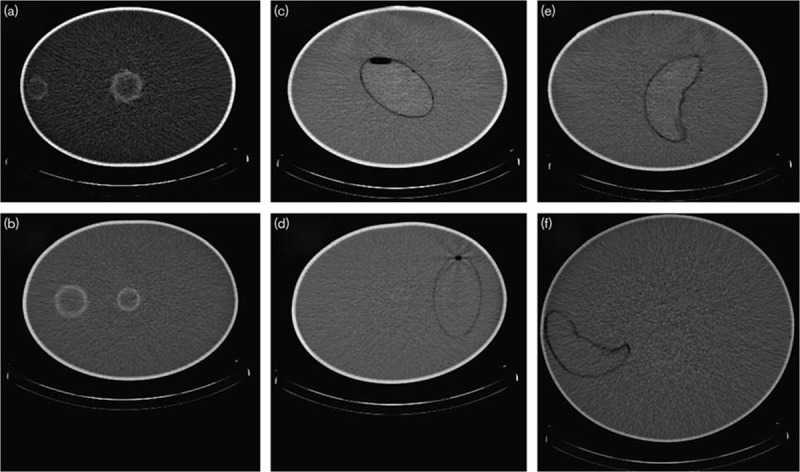
Computed tomography (CT) scans showing the positions of the inserts. (a) 113 ml sphere central, 16 ml sphere outer; (b) 16 ml sphere central, 113 ml sphere outer; (c) Cristy and Eckerman (C&E) spleen central; (d) C&E spleen outer; (e) patient spleen central; (f) patient spleen anatomical.

The inserts were mounted centrally and then radially displaced in an elliptical Jaszczak phantom apart from the patient spleen in its anatomical position which was mounted in a large elliptical phantom designed to mimic a patient body [5]. Figure 1 shows the positions of the inserts in the phantoms. The phantoms were filled with water and the inserts were filled with ^177^Lu-Dotatate solution. The sphere inserts were mounted in the same Jaszczak phantom for imaging, with one at the edge and one at the centre, as shown in Fig. 1a and b. The experimental images were reconstructed on each system with AC only and with both attenuation correction and triple energy window scatter correction (AC TEW) applied. The TEW scatter correction is applied to the projection set before reconstruction. The vendor-neutral system was capable of performing Monte Carlo based scatter correction, which was used as an additional reconstruction technique for the experimental images. The Monte Carlo scatter correction is performed before the forward projection step during the iterative reconstruction process.

In this work full Monte Carlo simulations of all the experimental scans were performed using GATE v8.0 [7]. Monte Carlo simulation provides the full history of all the detected particles, allowing any scattered events to be removed from the simulated projection images. A detailed model of the SPECT scanner was used, including the collimator, crystal and back compartment components. The phantoms were simulated with a voxelised geometry using the experimental CT scan as input. The insert sources were also voxelised and were defined by segmenting the experimental CT scans. The insert sources were also simulated in a vacuum to remove any impact of attenuation or AC and allow the impact of the DDSR to be assessed. The inserts were simulated containing the true activity in the insert, or 300 MBq if the true activity was greater than 300 MBq. The 300 MBq limit was imposed to constrain the running time of the simulations whilst providing adequate statistics. The two components of uncertainty considered were the statistical uncertainty on the number of counts in the VOI and the definition of the VOI. When using 300 MBq the statistical uncertainty on the number of counts in a VOI on the reconstructed SPECT image was ~ 0.04%, far less than the total uncertainty of around 1.5%. The majority of the uncertainty was due to the definition of the VOI. The uncertainty on the definition of the VOI was calculated by randomly perturbing the boundary of the VOI and observing the variation in counts for twenty different perturbations. This follows the ‘random’ method described by He and Frey [8]. The full imaging time was simulated for all the inserts. The simulated versions of the experimental projections were reconstructed on the vendor-specific system using AC TEW, and with all the scattered events removed. All scattered events were removed from the simulated projection images of the inserts in a vacuum. The projection images were then reconstructed with no corrections applied. The different reconstructed images are referred to as follows:

(1) Simulated projections in vacuum: Sim. vac.
(2) Experimental projections reconstructed with AC only: Exp. AC.
(3) Experimental projections reconstructed with AC and TEW scatter correction: Exp. AC TEW.
(4) Simulated projections reconstructed with AC and TEW scatter correction: Sim. AC TEW.
(5) Simulated projections with all scattered events, from any object, removed prior to reconstruction with AC: Sim. AC MCSC.
(6) Experimental projections reconstructed with AC and Monte Carlo based scatter correction: Exp. AC MCSC.

Experimental images were also acquired for the 113 ml sphere filled with ^99m^Tc solution, positioned as described for the ^177^Lu scans. SPECT scanners may be considered to be optimised for the use of ^99m^Tc. It is possible that the performance of the scanner or reconstruction is suboptimal for isotopes with emissions of different energies. The scans using ^99m^Tc were performed to examine the performance of the AC when the radionuclide for which the scanner may be considered to have been optimised was used. The attenuation of photons, and hence AC, is dependent on the gamma ray energy. Neither system specifies how the X-ray attenuation information from the CT scan is converted to the gamma ray energies. The ^99m^Tc images were reconstructed with only AC applied, and with each platforms default scatter correction used (ACSC, Double Energy Window for the vendor specific system and Monte Carlo based for the vendor neutral system). The acquisition settings for the scans using ^99m^Tc are shown in Table 2.

**Table 2:**
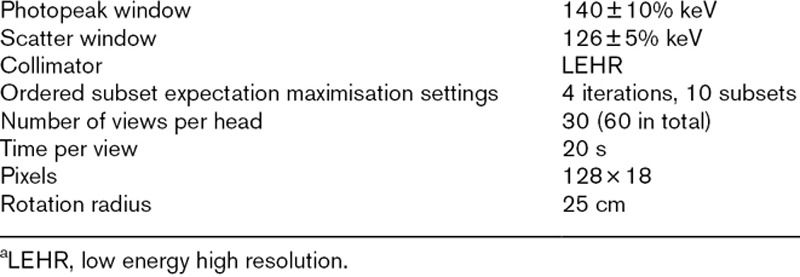
Acquisition settings for the single photon emission computed tomography scans of inserts filled with ^99m^Tc

A calibration factor can be defined which relates the count rate in a region to the activity in that region. The calibration factor is given by:


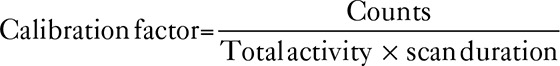
(1)

Calibration factors were calculated for all the inserts in all the reconstructed images. Whole image calibration factors were also calculated for each experimental and simulated scan. The insert calibration factors were calculated by outlining each insert on the CT scan, transferring the VOI to the corresponding SPECT image and calculating the total number of counts in the VOI. For the whole image calibration factors, all the counts in the reconstructed field of view were used. The calibration factor depends upon the scanner used, the collimator and the choice of reconstruction parameters and corrections. It is therefore sensitive to any changes in these [1]. It has previously been found that the camera sensitivity is within 6% for a point source in air and a spherical source surrounded by background activity [3].

## Results

**Fig. 2:**
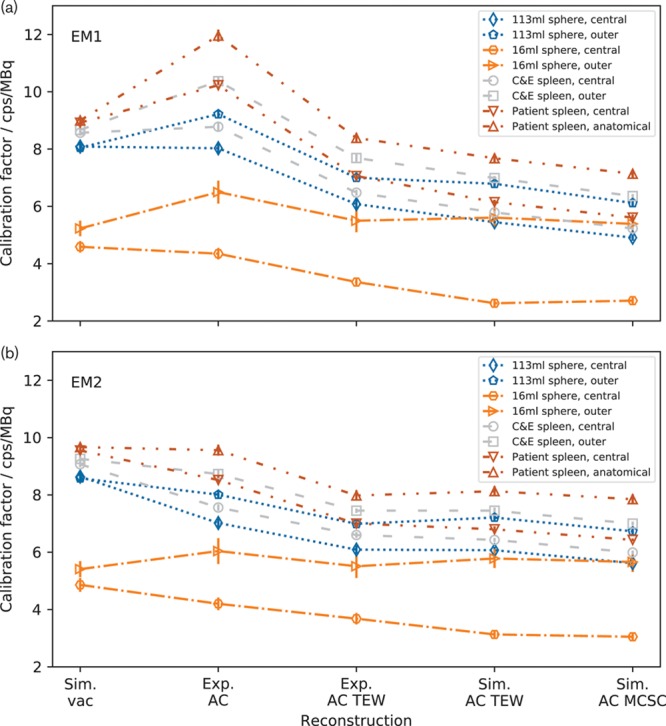
Calibration factors for all the inserts reconstructed using different corrections using the vendor-specific system for (a) the EM1 energy window and (b) the EM2 energy window. Note that the calibration factors for each organ are consistent in a vacuum and diverge when Exp. attenuation correction (AC) is used. The dashed lines are added to guide the eye and do not indicate a continuity of data. The central Cristy and Eckerman (C&E) spleen data are from Robinson *et al.* [4] and the patient spleen data are from Price *et al.* [5].

Figure 2 shows insert calibration factors for the images reconstructed using the vendor-specific system. It can be seen that the calibration factors for each insert are consistent when the insert is radially displaced in vacuum for both the EM1 and EM2 windows. The calibration factors differ significantly by more than 3 standard deviations (SD) when AC is applied to experimental data, that is, the phantom containing water. The calibration factor for inserts near the edge of the phantom is higher than those for the inserts positioned centrally. The calibration factors for EM1 increase relative to those for vacuum when AC is applied and those for EM2 decrease. This is due to the difference in the proportion of scattered events in the energy windows. The behaviour of the values is similar when scatter correction is used. If AC and TEW scatter correction work correctly, they should not introduce a positional dependence of calibration factor. Applying AC TEW to the experimental data results in a reduction of calibration factor but the values remain significantly different. The calibration factors calculated from the simulated ACSC data are consistent with experiment within 2 SD for EM2, while those for EM1 are lower by between 8 and 14%. The simulated calibration factors for each insert in the two positions are not significantly more different than the experimental values. The use of Sim. AC MCSC data gives a small reduction in calibration factor but does not improve the consistency between the values.

**Fig. 3:**
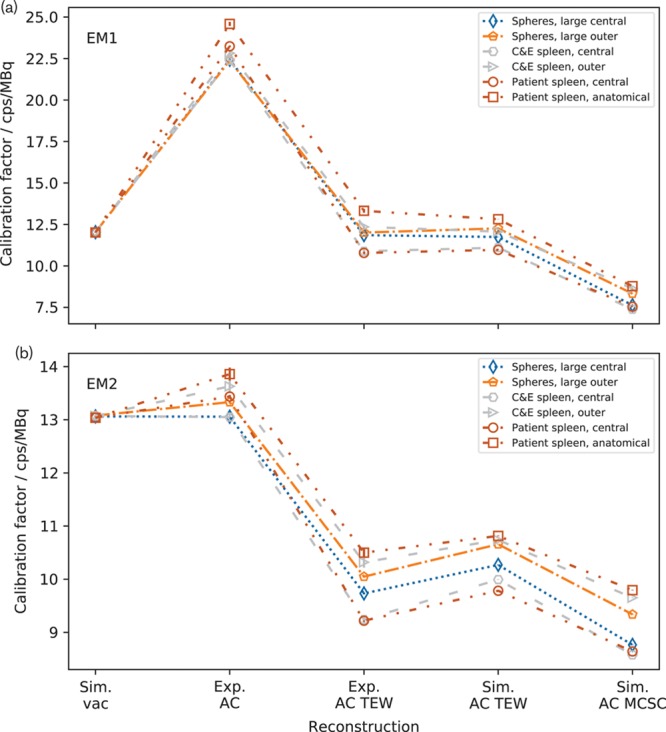
Whole image calibration factors for all the inserts reconstructed using different corrections using the vendor-specific system for (a) the EM1 energy window and (b) the EM2 energy window. Note that all the calibration factors for the inserts in the vacuum are consistent and then diverge when Exp. attenuation correction (AC) is used. The spheres were imaged in the same phantom so are cannot be shown separately in this plot. The dashed lines are added to guide the eye. The central Cristy and Eckerman (C&E) spleen data are from Robinson *et al.* [4] and the patient spleen data are from Price *et al.* [5].

Figure 3 shows the calibration factors for the whole field of view and for the different inserts and positions, using the vendor-specific system. When the inserts were simulated in vacuum the calibration factors were within a range of 0.03%. Again, when AC is applied to experimental data the calibration factors differ with inserts nearer the edge of the phantom having higher calibration factors, but a smaller spread. The discrepancy remains when AC TEW and Sim. AC MCSC is applied. There is a large increase in the calibration factor for the EM1 data when only AC is used.

**Fig. 4:**
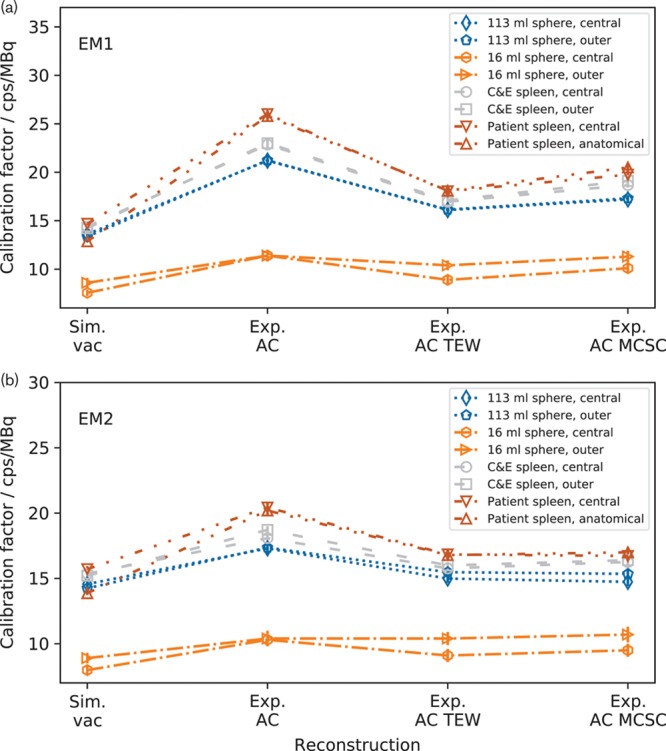
Calibration factors for images reconstructed using the vendor-neutral system for (a) the EM1 energy window and (b) for the EM2 energy window. Note that the calibration factors for each insert are consistent both when Exp. attenuation correction (AC), Exp. AC triple energy window (TEW) and Exp. AC MCSC is applied. The dashed lines are added to guide the eye and do not show a continuity of data.

Figure 4 shows insert calibration factors for images reconstructed using the vendor-neutral system. For both energy windows, the calibration factors for a given insert are consistent when AC is applied. The use of AC TEW reduces the calibration factors but the values remain consistent. Similarly, the use of Exp. AC MCSC changes the absolute value of the calibration factors but those for each insert remain consistent. Table 3 shows a comparison of the percentage differences between the calibration factors for each insert in the two positions, reconstructed using the vendor neutral and vendor specific systems. On the vendor-specific system, the differences are between 12 and 14% for the inserts apart from the 16 ml sphere. For the vendor neutral system, the differences are between 0 and 4%, apart from the 16 ml sphere, and all are consistent with zero.

**Table 3:**
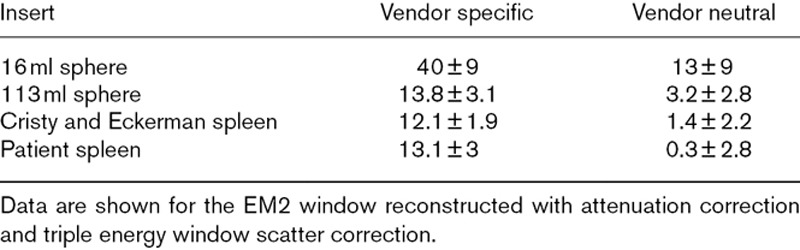
The percentage differences between the calibration factors for the inserts in the two positions

**Fig. 5:**
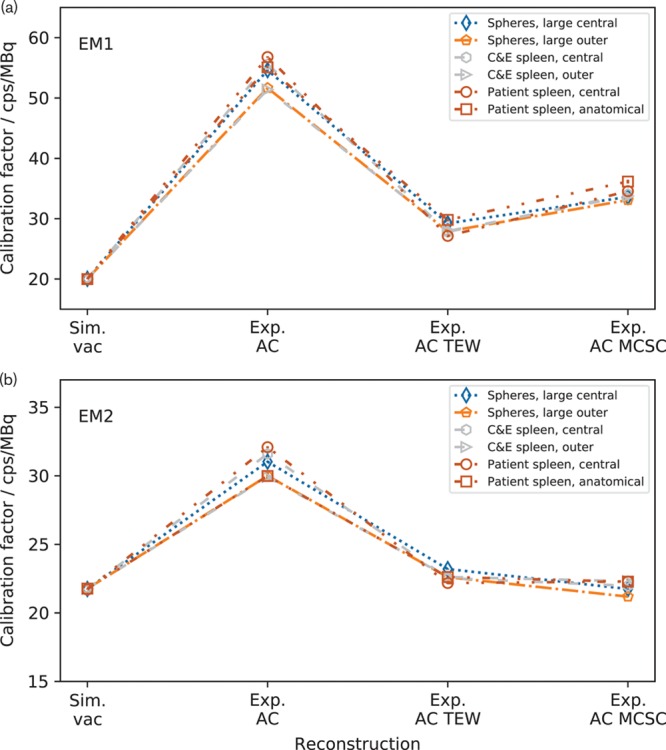
Whole image calibration factors for images reconstructed using the vendor-neutral system for (a) the EM1 energy window and (b) for the EM2 energy window. The calibration factors diverge when Exp. attenuation correction (AC) is applied and converge with Exp. AC triple energy window (TEW) or Exp. AC MCSC. The dashed lines are added to guide the eye and do not show a continuity of data.

Figure 5 shows whole image calibration factors for the images reconstructed on the vendor-neutral system. For each insert, the calibration factors show a very small difference when AC is applied which is reduced when AC TEW is applied. Using Exp. AC MCSC changes the values of the calibration factors but does not change their consistency.

**Fig. 6:**
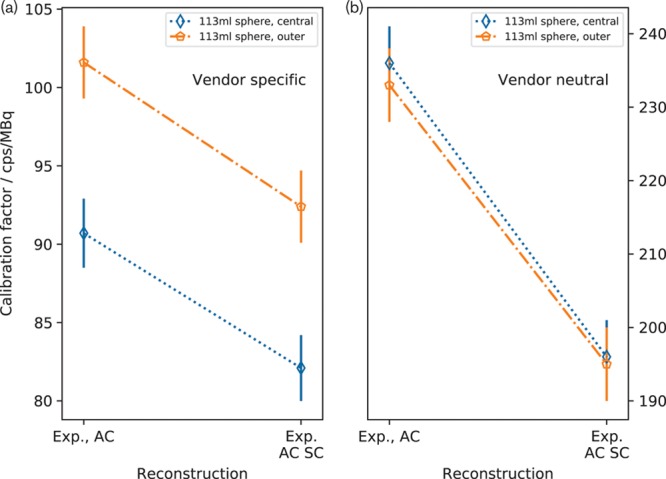
Volume of interest (VOI) calibration factors for the experimental ^99m^Tc scans. The calibration factors for the vendor-neutral system (b) agree much more closely than those for the vendor-specific system (a).

Figure 6 shows insert calibration factors for the scans of the sphere filled with ^99m^Tc solution. The calibration factors for the vendor-neutral system are in much closer agreement than those for the vendor-specific system when both AC and ACSC are applied.

The vendor-specific system outputs the attenuation map used during the reconstruction, but the pixel data is stored in arbitrary units. The pixel values for water are ~162 for EM1 and ~132 for EM2. The percentage difference between the pixel values for water for both energy windows is approximately the same as the percentage difference in photon cross-sections for photons of 114 and 208 keV.This suggests that the measured attenuation coefficients from the CT scan are successfully converted to those for photons with energies of 114 and 208 keV on this system.

## Discussion

The consistency between the experimental and simulated AC TEW data validates the simulation by demonstrating that experimental data can be reproduced. This consistency also demonstrates that the radial dependence is not due to PMT signal processing as this processing is not modelled in the Monte Carlo simulation. If the dependence was due to the signal processing it would not be visible in simulated data. The electronics in the detector can, therefore, be ruled out as the cause of the positional dependence.

The lack of large radial dependence in the simulated images in vacuum demonstrates that the inserts are not sufficiently displaced from the centre of rotation for the DDSR to have a major impact on calibration factor. The differences between the VOI calibration factors for the inserts in vacuum are probably due to the differences in partial volume effects for the inserts during imaging and segmentation [1]. The agreement of the whole image calibration factors demonstrates that the differences in VOI calibration factors in vacuum are not due to changes in the sensitivity of the detector. For inserts displaced up to the distances examined, the DDSR is not a significant cause of the positional dependence of calibration factor.

The use of AC on the vendor-specific system introduces a radial dependence, which is not improved by the application of TEW scatter correction to experimental and simulated images. The removal of all scattered photons using the full Monte Carlo simulation in GATE also does not remove the radial dependence. The lack of convergence when Sim. AC MCSC is used demonstrates that the TEW scatter correction is working as expected. However, when a different vendor neutral reconstruction algorithm is used, with a different AC method built in, the radial dependence is absent. The vendor-neutral system does not introduce a radial dependence when Exp. AC TEW or Exp. AC MCSC is applied. Neither system allows the user to interrogate how the CT data is interpreted during the reconstruction, but the vendor-neutral system does allow the user to input attenuation coefficients read from a CT phantom. The proportional difference between the attenuation map values for the EM1 and EM2 energy windows on the vendor-specific system suggest that the CT numbers are converted to attenuation coefficients correctly. The ^99m^Tc images reconstructed using the vendor-specific system display the positional dependence as for ^177^Lu. The vendor-neutral system shows the same lack of dependence. This demonstrates that the positional dependence is still present when a different isotope is used.

The positional dependence introduces a systematic increase of around 12% to the calibration factor of inserts positioned near the edge of a phantom. This is similar to the uncertainty on cumulated activity found in recent EANM guidance on uncertainty analysis [9].

These data have demonstrated that the different methods of estimating AC inbuilt in the reconstruction algorithms do not necessarily maintain a constant calibration factor throughout an attenuating medium. The DDSR of the SPECT camera and corrections for scattered photons have been demonstrated to not account for the positional dependence. This effect leads to a higher calibration factor near the edge of the medium for one system. It should be noted that a positional dependence was observed for different systems by Wevrett *et al.* [2]. For accurate dosimetry calculations, this work indicates that users of reconstruction systems should check the behaviour of their own systems and determine whether any correction for the position, such as using position-dependent calibration factors, is needed.

## Acknowledgements

This work was supported by the Science and Technology Facilities Council (grant numbers ST/M003302/1 and ST/P000150/1) and HERMES Medical Solutions Ltd.

## Conflicts of interest

Emlyn Price is partially funded by Hermes Medical Solutions Ltd. For the remaining authors there are no conflicts of interest.
